# 2-Phenylethylamine (PEA) Ameliorates Corticosterone-Induced Depression-Like Phenotype via the BDNF/TrkB/CREB Signaling Pathway

**DOI:** 10.3390/ijms21239103

**Published:** 2020-11-30

**Authors:** Young-Ju Lee, Hye Ryeong Kim, Chang Youn Lee, Sung-Ae Hyun, Moon Yi Ko, Byoung-Seok Lee, Dae Youn Hwang, Minhan Ka

**Affiliations:** 1 Pharmacology and Drug Abuse Group, Convergence Toxicology Research Division, Korea Institute of Toxicology, KRICT, Daejeon 34114, Korea; youngju.lee@kitox.re.kr (Y.-J.L.); hye9314@kbri.re.kr (H.R.K.); changyeon.lee@kitox.re.kr (C.Y.L.); sahyun@kitox.re.kr (S.-A.H.); moonyi.ko@kitox.re.kr (M.Y.K.); 2 Department of Biomaterials Science, College of Natural Resources and Life Science/Life and Industry Convergence Research Institute, Pusan National University, Miryang 50463, Korea; dyhwang@pusan.ac.kr; 3 Laboratory Animal Center, Korea Brain Research Institute, Daegu 61062, Korea; 4 Department of Advanced Toxicology Research, Korea Institute of Toxicology, KRICT, Daejeon 34114, Korea; bslee@kitox.re.kr

**Keywords:** 2-Phenylethylamine (PEA), depression, corticosterone, depression-like behavior, BDNF/TrkB/CREB signaling

## Abstract

Depression is a serious medical illness that is one of the most prevalent psychiatric disorders. Corticosterone (CORT) increases depression-like behavior, with some effects on anxiety-like behavior. 2-Phenethylamine (PEA) is a monoamine alkaloid that acts as a central nervous system stimulant in humans. Here, we show that PEA exerts antidepressant effects by modulating the Brain-derived neurotrophic factor (BDNF)/tropomyosin receptor kinase B (TrkB)/cAMP response element binding protein (CREB) signaling pathway in CORT-induced depression. To investigate the potential effects of PEA on CORT-induced depression, we first treated CORT (50 μM)-induced hippocampal neurons with 100 μM PEA for 24 h. We found that treatment with CORT altered dendritic spine architecture; however, treatment with PEA rescued dendritic spine formation via regulation of BDNF/TrkB/CREB signaling. Next, we used a mouse model of CORT-induced depression. Mice were treated with CORT (20 mg/kg) for 21 days, followed by assessments of a battery of depression-like behaviors. During the final four days of CORT exposure, the mice were treated with PEA (50 mg/kg). We found that CORT injection promoted depression-like behavior and significantly decreased BDNF and TrkB expression in the hippocampus. However, treatment with PEA significantly ameliorated the behavioral and biochemical changes induced by CORT. Our findings reveal that PEA exerts antidepressant effects by modulating the BDNF/TrkB/CREB signaling pathway in a mouse model of CORT-induced depression.

## 1. Introduction

Major depressive disorder (MDD) is a highly prevalent neuropsychiatric disorder and is a major public health concern [1]. Chronic stress is an important risk factor for the development of major depression [2], and stress-induced dysregulation of the hypothalamic-pituitary-adrenal (HPA) axis is involved in the pathogenesis of depression [3,4]. Dysregulation of the HPA axis is a common finding in depression, which results in increased circulating glucocorticoid levels in blood [5] and, as a result, leads to oxidative stress [6,7], glutamatergic excitotoxicity [8,9] and neuroinflammation [10]. Chronic exposure of rodents to corticosterone (CORT) is widely used to induce depression-like behavior and neurochemical changes associated with MDD symptoms, including anhedonic and anxiety behaviors [11,12] and decreased levels of synaptic-related proteins [13,14]. In addition, a chronic CORT-induced rodent model is appropriate for evaluating the efficacy of antidepressant drugs and exploring the mechanism of action of antidepressants [15].

Brain-derived neurotrophic factor (BDNF) is a well-known growth factor that plays a critical role in several aspects of brain function, including neurogenesis, neuronal survival, neuronal maturation, synapse formation and synaptic plasticity in the brain [16,17]. BDNF has been implicated in neuropsychiatric disorders, including schizophrenia, intellectual disability, autism, and mood disorders. BDNF and its mechanisms are also therapeutic targets of pharmacological agents that are currently used to treat these diseases, such as antidepressants and antipsychotics [18]. For example, AMPK activation produces anti-depressant effects, which are mediated hippocampal neurogenesis via PKCζ/NF-κB/BDNF/TrkB/CREB signaling in neurons [19]. *Asparagus cochinchinensis* extract exerts antidepressant effects, possibly via modulation of the BDNF-TrkB pathway, in a rat model of menopausal depression [20]. BDNF binds with its receptor tropomyosin receptor kinase B (TrkB) and phosphorylated TrkB can activate the Ras/ERK, PLC-γ, and phosphatidylinositol 3-kinase (PI3K)/AKT pathways [21,22]. Activated PLC-γ directly induces Ca^2+^/calmodulin-dependent kinase (CaMKII) [23,24] and activated CaMKII can activate BDNF expression by activating the transcription factor CREB [25,26]. Recent animal studies have shown that chronic exposure to stressors significantly decreases BDNF expression in the hippocampus [27,28]; moreover, chronic CORT exposure reduces BDNF mRNA and protein expression in the hippocampus [29]. Several lines of evidence support that BDNF plays an essential role in antidepressants [30]. Chronic antidepressant treatments activates BDNF mRNA and protein expression in distinct regions of the brain [31], and pretreatment with antidepressants prevents stress-induced decreases in hippocampal BDNF expression [32]. In addition, the TrkB receptor has also been shown to be related to antidepressant treatment [33].

2-Phenethylamine (PEA) is an endogenous natural monoamine alkaloid that has stimulant effects that lead to the release of neurotransmitters, including dopamine and serotonin [34,35]. PEA binds to a novel trace amine-associated receptor (TAAR), which leads to the inhibition of dopamine, serotonin and norepinephrine reuptake [36]. Genetic deletion of TAAR1 induces increased sensitivity to psychostimulants and is associated with schizophrenia [37,38]. PEA improves mood in patients with depression who are treated with a selective monoamine oxidase B inhibitor [39]. However, the antidepressant effects of PEA are not fully understood. In this study, we determined the antidepressant mechanisms of PEA in CORT-induced mice. We report that treatment with PEA can ameliorate depression-like behavior and biochemical responses in mice with CORT-induced depression. Our findings indicate the potential therapeutic effects of PEA for treating depression and suggest a mechanism by which PEA may alleviate depression-like symptoms.

## 2. Results

### 2.1. PEA Restores Dendritic Spines Formation in CORT-Induced Hippocampal Neurons

**Figure 1:**
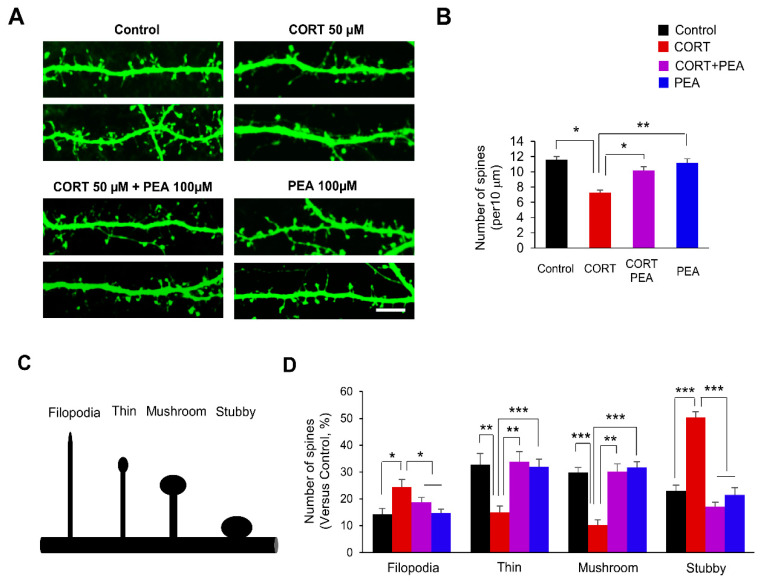
Treatment with 2-Phenethylamine (PEA) restores dendritic spine formation in corticosterone (CORT)-induced hippocampal neurons. (**A**) PEA restores dendritic spine density in CORT-induced hippocampal neurons. Cultured hippocampal neurons were isolated from E18 embryos, cultured and transfected with a GFP plasmid at DIV 10. After 4 days, neuronal cultures were administrated with the CORT (50 μM) for 24 h followed by treatment with PEA (100 μM) for 24 h. Scale bar, 10 μm. (**B**) Quantification of the number of dendritic spines in each condition. *n* = 15 neurons from three independent cultures using three mice for each condition. Statistical significance was determined by two-way ANOVA with Bonferroni correction test. Data are shown as relative changes versus controls. * *p* < 0.05, ** *p* < 0.01 (**C**) Different types of dendritic spines (Filopodia, thin, mushroom, and stubby). (**D**) PEA restores dendritic spine morphology in CORT-induced hippocampal neurons. *n* = 10 cultured cortical neurons and 300 dendritic spines for each condition. Statistical significance was determined by two-way ANOVA with Bonferroni correction test. Data are shown as relative changes versus controls. * *p* < 0.05, ** *p* < 0.01, *** *p* < 0.001.

CORT reduces dendritic spines in developing hippocampal neurons [40]. Thus, we investigated whether PEA affected dendritic spine formation in CORT-induced hippocampal neurons. Based on the concentration-dependent effects of CORT and PEA on cytotoxicity in hippocampal neurons (Supplemental Figure S1A,B), we cultured hippocampal neurons from embryonic day 18 (E18) mice for 10 days and transfected them with a plasmid encoding green fluorescent protein (GFP). After 4 days, the neuronal cultures were treated with CORT for 24 h, followed by treatment with PEA for 24 h, and then dendritic spines were assessed using confocal microscopy. As expected, CORT exposure decreased the number of dendritic spines by 37% compared with that of control neurons (Figure 1A,B). However, treatment with PEA suppressed the CORT-induced decrease in dendritic spines (Figure 1A,B).

Activity-dependent morphological changes in dendritic spines play essential roles in neural plasticity [41]. Next, we sought to investigate whether PEA could rescue the morphological changes in CORT-induced dendritic spines. Dendritic spines can be classified into categories such as filopodia and thin, mushroom or stubby spines [42]. Dendritic spines can be classified into categories such as filopodia, thin, mushroom or stubby spines. Filopodia are typically longer (>2 μm) and normally have no clear head; thin spines have a thin, long neck (>1 μm) and small heads; mushroom spines have a short and narrow neck (<1 μm) and a large head (>0.6 μm), whereas the stubby spines have a head but no neck (Figure 1C). CORT-induced hippocampal neurons displayed significant reductions in mushroom, thin and stubby spines compared with those of control neurons (Figure 1A). The numbers of thin and mushroom spines decreased by 54% and 66%, respectively, in CORT-induced neurons (Figure 1A,D). However, the numbers of filopodia and stubby spines increased by 71% and 118%, respectively, in CORT-induced neurons. Interestingly, treatment with PEA suppressed the morphological changes in the dendritic spines of CORT-induced neurons (Figure 1A,D). Taken together, these results show that treatment with PEA rescues CORT-induced dendritic spine malformation.

### 2.2. PEA Induces Excitatory Synapses by Regulating BDNF/TrkB/CREB Signaling in CORT-Induced Hippocampal Neurons

**Figure 2:**
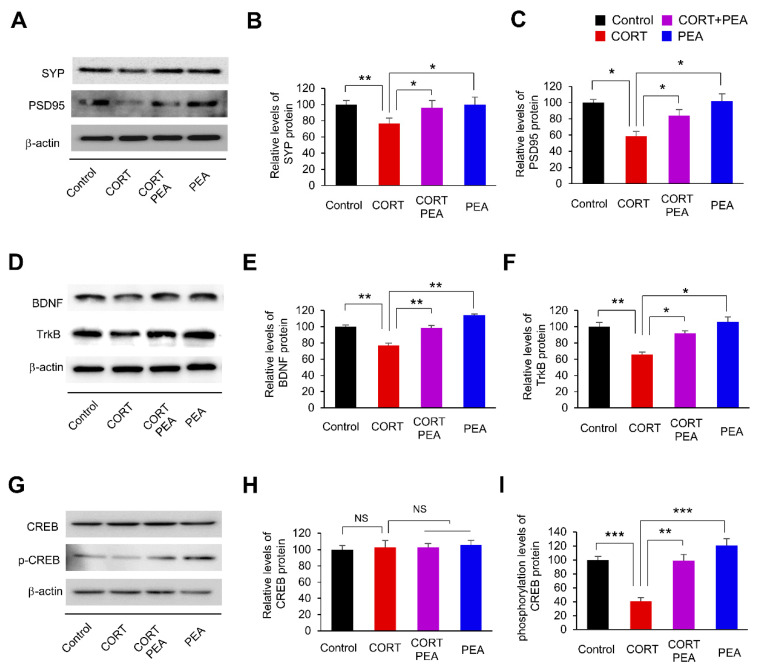
Treatment with PEA restores excitatory synaptic activity by regulation of Brain-derived neurotrophic factor (BDNF)/tropomyosin receptor kinase B (TrkB)/cAMP response element binding protein (CREB) signaling in CORT-induced hippocampal neurons. (**A**) PEA rescues presynaptic and postsynaptic molecules in CORT-induced hippocampal neurons. Cellular lysates were isolated from cultured hippocampal neurons. Neuronal cultures were administrated with the CORT (50 μM) for 24 h followed by treatment with PEA (100 μM) for 24 h. Western blotting was performed with an SYP and a PSD95 antibodies. (**B**,**C**) Quantification of protein levels shown in (**A**). The levels of protein were normalized to β-actin expression. *n* = 3 independent cultures using three mice. Statistical significance was determined by two-way ANOVA with Bonferroni correction test. Data are shown as relative changes versus controls. * *p* < 0.05, ** *p* < 0.01. (**D**) PEA restores the levels of BDNF and TrkB in CORT-induced hippocampal neurons. Western blotting was performed with a BDNF and TrkB antibodies. (**E**,**F**) Quantification of protein levels shown in (**D**). The levels of protein were normalized to β-actin expression. *n* = 3 independent cultures using three mice. Statistical significance was determined by two-way ANOVA with Bonferroni correction test. Data are shown as relative changes versus controls. * *p* < 0.05, ** *p* < 0.01. (**G**) PEA restores the phosphorylation level of CREB in CORT-induced hippocampal neurons. Western blotting was performed with a CREB and p-CREB antibodies. (**H**,**I**) Quantification of protein levels shown in (**G**). The levels of protein were normalized to β-actin expression. *n* = 3 independent cultures using three mice. Statistical significance was determined by two-way ANOVA with Bonferroni correction test. Data are shown as relative changes versus controls. NS: not significant, ** *p* < 0.01, *** *p* < 0.001.

Having determined that PEA restores dendritic spine density and morphology in CORT-induced hippocampal neurons (Figure 1), we next investigated whether treatment with PEA could also lead to alterations in excitatory synapses in CORT-induced hippocampal neurons. We cultured hippocampal neurons from E18 mice for 14 days and exposed the neurons to CORT for 24 h, followed by PEA treatment for 24 h. Then, we assessed the levels of excitatory synaptic markers, such as synaptophysin (SYP, presynaptic) and PSD95 (postsynaptic), in the lysates of cultured hippocampal neurons. We found that the levels of SYP and PSD95 decreased by 23% and 42%, respectively, in CORT-induced hippocampal neurons (Figure 2A–C). However, treatment with PEA rescued SYP and PSD95 expression (Figure 2A–C). These findings demonstrate that CORT alters excitatory synapses in hippocampal neurons and suggests that there is an underlying cause of abnormal synaptic functions. In addition, PEA restores CORT-induced inhibition of excitatory synapses in hippocampal neurons.

Previously, it was reported that CORT regulates the expression of BDNF and TrkB [29,43]. Thus, we investigated whether PEA affected BDNF and TrkB expression levels in CORT-induced hippocampal neurons. We found that the levels of BDNF and TrkB decreased by 24% and 34%, respectively, in CORT-induced hippocampal neurons compared with control neurons (Figure 2D–F). However, treatment with PEA rescued BDNF and TrkB expression in CORT-induced hippocampal neurons (Figure 2D–F). These results show that treatment with PEA rescues CORT-induced inhibition of the BDNF/TrkB signaling pathway. CREB is a transcription factor that regulates the synthesis of synaptic proteins [44,45]. We investigated whether PEA affected the activation of CREB in CORT-induced hippocampal neurons. Hippocampal neurons were treated with PEA for 24 h, followed by CORT exposure for 24 h. Cellular lysates were subjected to western blotting to measure phosphorylated CREB levels. We found that the phosphorylation level of CREB was decreased by 61% in CORT-induced hippocampal neurons compared with control neurons (Figure 2G–I). However, treatment with PEA restored CREB phosphorylation in CORT-induced hippocampal neurons (Figure 2G–I). These results show that treatment with PEA restores excitatory synaptic proteins by activating BDNF/TrkB/CREB signaling in CORT-induced hippocampal neurons.

### 2.3. PEA Promotes Excitatory Synapses by Regulating BDNF/TrkB/CREB Signaling in the CORT-Induced Hippocampus

**Figure 3:**
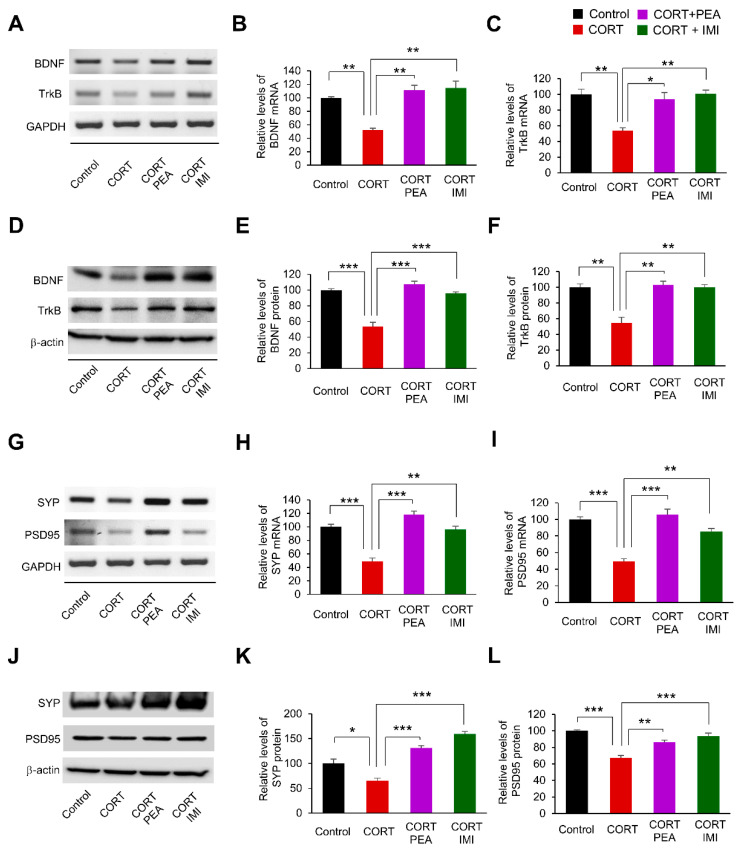
Treatment with PEA activates BDNF/TrkB signaling in CORT-induced mice. (**A**) PEA restores the mRNA levels of BDNF and TrkB in CORT-induced mice. Mice were exposed with either CORT (20 mg/kg) or saline (control) once a day for 21 days. After 17 days of initial administration, the mice received daily treatment of PEA (50 mg/kg) or imipramine (10 mg/kg) during an additional 4 days of CORT exposure. The mRNA levels of BDNF and TrkB were assessed by RT-PCR in hippocampus. (**B**,**C**) Quantification of mRNA levels shown in (**A**). The levels of mRNA were normalized to GAPDH transcription. *n* = 3 independent tissue using three mice. Statistical significance was determined by two-way ANOVA with Bonferroni correction test. Data are shown as relative changes versus controls. * *p* < 0.05, ** *p* < 0.01. (**D**) PEA restores the levels of BDNF and TrkB proteins in CORT-induced mice. (**E**,**F**) Quantification of protein levels shown in (**D**). The levels of protein were normalized to β-actin expression. *n* = 3 independent tissue using three mice. Statistical significance was determined by two-way ANOVA with Bonferroni correction test. Data are shown as relative changes versus controls. ** *p* < 0.01, *** *p* < 0.001. (**G**) PEA rescues the mRNA levels of PSD95 and SYP in CORT-induced mice. (**H**,**I**) Quantification of mRNA levels shown in (**G**). The levels of mRNA were normalized to GAPDH transcription. *n* = 3 independent cultures using three mice. Statistical significance was determined by two-way ANOVA with Bonferroni correction test. Data are shown as relative changes versus controls. ** *p* < 0.01, *** *p* < 0.001. (**J**) PEA restores the levels of PSD95 and SYP proteins in CORT-induced mice. (**K,L**) Quantification of protein levels shown in (**J**). The levels of protein were normalized to β-actin expression. *n* = 3 independent tissue using three mice. Statistical significance was determined by two-way ANOVA with Bonferroni correction test. Data are shown as relative changes versus controls. * *p* < 0.05, ** *p* < 0.01, *** *p* < 0.001.

Based on the effects of PEA on BDNF and TrkB expression in CORT-induced hippocampal neurons, we sought to examine whether PEA administration in vivo would have similar effects on the expression of BDNF and TrkB. Thus, mice were exposed to either CORT (20 mg/kg) or saline (control) once a day for 21 days. After 17 days of administration, the remaining mice received daily treatment with PEA (50 mg/kg) or imipramine (10 mg/kg) during an additional 4 days of CORT exposure. The positive control, imipramine is a tricyclic antidepressant and dose-dependently increased BDNF mRNA expression [46]. Using RT-PCR, we first measured the mRNA levels of BDNF and TrkB in hippocampal lysates. We found that BDNF and TrkB mRNA levels were reduced by 48% and 46%, respectively, in CORT-induced mice compared with controls (Figure 3A–C). However, treatment with either PEA or imipramine restored BDNF and TrkB mRNA levels (Figure 3A–C). Similarly, we found that the protein levels of BDNF and TrkB decreased by 47% and 45%, respectively, in CORT-induced mice compared with controls (Figure 3D–F). However, treatment with either PEA or imipramine rescued BDNF and TrkB protein expression in CORT-induced mice (Figure 3D–F). In parallel, we determined the levels of excitatory synaptic markers, such as SYP and PSD95, in hippocampal lysates. The mRNA levels of SYP and PSD95 decreased by 51% and 50%, respectively, in CORT-induced hippocampal lysates compared with controls (Figure 3G–I). However, treatment with either PEA or imipramine restored excitatory synaptic marker transcription to baseline levels (Figure 3G–I). We also assessed the protein levels of SYP and PSD95. The levels of SYP and PSD95 decreased by 47% and 33%, respectively, in CORT-induced mice (Figure 3J–L). However, treatment with either PEA or imipramine partially restored SYP and PSD95 expression (Figure 3J–L). These results confirm that PEA alters excitatory synapses by regulating BDNF/TrkB/CREB signaling in the CORT-induced hippocampus.

### 2.4. PEA Ameliorates Depression-Like Behavior in CORT-Induced Mice

**Figure 4:**
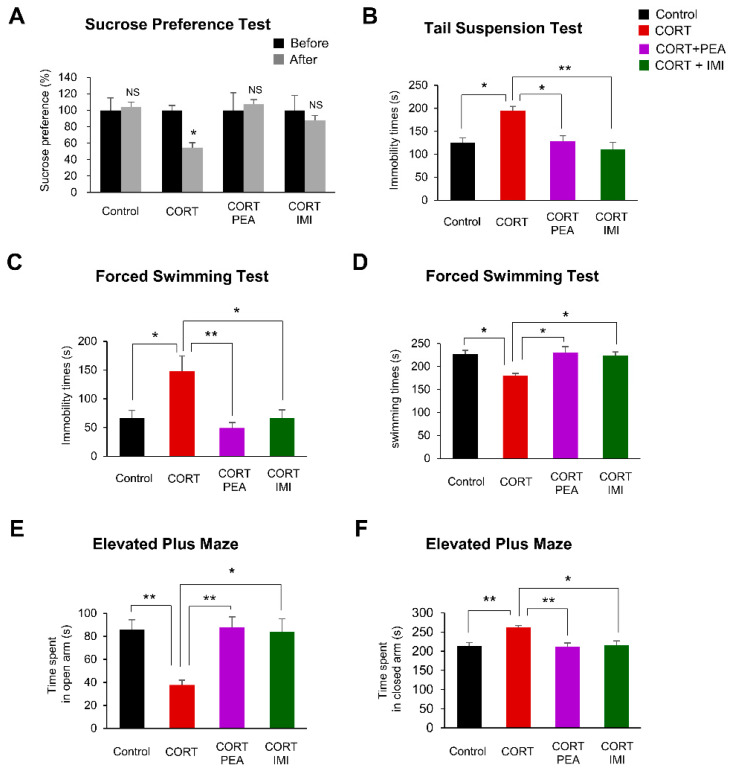
Treatment with PEA rescues depression-like behaviors in CORT induced mice. (**A**) The anhedonic behavior of mice during sucrose preference test (SPT). The rate of sucrose intake before CORT exposure and after treatment with PEA. PEA restores the sucrose preference in CORT-induced mice. *n* = 7 mice for each experimental group. Statistical significance was determined by two-way ANOVA with Bonferroni correction test. Data are shown as relative changes versus controls. NS; not significant, * *p* < 0.05. (**B**) The depression-like behavior of mice during tail suspension test (TST). PEA restores the immobility times in CORT-induced mice. *n* = 7 mice for each experimental group. Statistical significance was determined by two-way ANOVA with Bonferroni correction test. Data are shown as relative changes versus controls. * *p* < 0.05, ** *p* < 0.01. (**C**,**D**) The depression-like behavior of mice during forced swimming test (FST). The time of immobility (**C**) and the time of swimming (**D**). PEA administration decreased immobility times and increased swimming times in CORT-induced mice. *n* = 7 mice for each experimental group. Statistical significance was determined by two-way ANOVA with Bonferroni correction test. Data are shown as relative changes versus controls. * *p* < 0.05, ** *p* < 0.01. (**E**,**F**) The anxiety-like behavior of mice during elevated plus maze (EPM). The spent time in open arm (**E**) and the spent time in closed arm (**F**). PEA administration decreased the spent time in open arm and increased the spent time in closed arm in CORT-induced mice. *n* = 7 mice for each experimental group. Statistical significance was determined by two-way ANOVA with Bonferroni correction test. Data are shown as relative changes versus controls. * *p* < 0.05, ** *p* < 0.01.

To measure the efficacy of PEA in treating CORT-induced depression-like behavior, we first assessed the anhedonic state of mice by the sucrose preference test (SPT). We found that CORT led to a 44% reduction in sucrose preference, but treatment with PEA or imipramine successfully restored sucrose preference to control levels (Figure 4A). Next, we investigated whether PEA affected depression-like behavior in CORT-induced mice by the tail suspension test (TST). We found that the immobility time was significantly increased by 55% in CORT-induced mice compared with control mice (Figure 4B). However, treatment with either PEA or imipramine reversed this depression-like phenotype to normal levels (Figure 4B).

To confirm the observed antidepressant effects of PEA, we analyzed the immobility and swimming time of mice exposed to CORT following treatment with PEA or imipramine with a forced swimming test (FST). CORT led to a 123% increase in immobility time and a corresponding 21% decrease in swimming time. Treatment with either PEA or imipramine reversed this depression-like phenotype to normal levels (Figure 4C,D). These results suggest that PEA is as effective as imipramine in treating depression in a mouse model.

Finally, to investigate the effects of PEA on anxiety-like behavior in CORT-induced mice, we analyzed the time the mice spent in the open or closed arm in the elevated plus maze (EPM). We found that the time spent in the open arm was decreased by 56% in CORT-induced mice compared with control mice (Figure 4E). In contrast, the time CORT-induced mice spent in the closed arm was increased by 23% compared with that of control mice (Figure 4F). Treatment with either PEA or imipramine reversed this anxiety-like phenotype to baseline levels (Figure 4E,F). Taken together, these results suggest that PEA has anxiolytic—in addition to antidepressant—effects on CORT-induced mice.

## 3. Discussion

**Figure 5:**
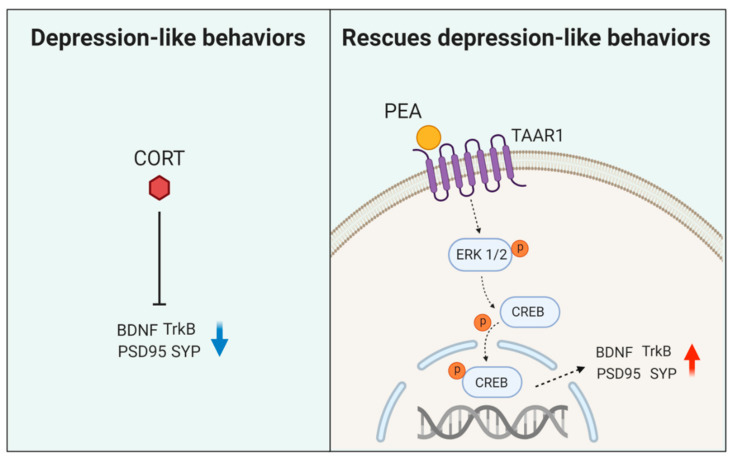
A schematic model illustrating an effect of PEA on CORT-induced depression-like mice model. PEA activates BDNF/Trk-B signaling, leading to the upregulation of excitatory synaptic function in CORT-induced mice. Activation of BDNF/Trk-B signaling rescues abnormal synaptic architecture and behaviors in CORT-induced depression-like mice.

In this study, we provide evidence that CORT induces depression-like behaviors and several cellular and molecular hallmarks of depression and that treatment with PEA ameliorates these symptoms. In hippocampal neurons, the inhibition of excitatory synaptic protein expression through CREB inactivation plays an important role in CORT-induced depression-like behaviors. PEA ameliorates CORT-induced depression-like behaviors by activating BDNF/TrkB/CREB signaling (Figure 5). Our results provide novel insight into the antidepressant properties of PEA. Understanding the mechanisms of the CORT-induced depression model could have implications for the future development of antidepressant therapeutic targets.

Depression is closely related to changes in dendritic spine density and morphology. Chronic mild stress is a widely used animal model of depression [47] and induces dendritic spine atrophy and loss in the excitatory neurons of the hippocampus and prefrontal cortex (PFC). Moreover, neurons of the amygdala and nucleus accumbens show increases in dendritic spine density [48]. Moreover, CORT reduces dendritic complexity in hippocampal CA1 neurons [40]. We found that CORT administration modified dendritic spine density and morphology, which was related to synaptic function [49,50]. CORT exposure increased filopodia and stubby spines and decreased thin and mushroom spines in hippocampal neurons. Consistently, previous studies reported that postpartum CORT administration reduced dendritic complexity and increased mushroom spines in hippocampal neurons [51]. Alterations in neuronal plasticity induced by CORT could thus involve a combination of factors including cell specificity, dendritic spine morphology and synaptic strength. We also found that treatment with PEA restored dendritic spine formation in CORT-induced hippocampal neurons. PEA, an endogenous trace amine, has been shown to alleviate depression in 60% of patients. However, the underlying mechanisms of the antidepressant effect of PEA remain poorly understood. Our findings suggest that PEA treatment may increase the level of N-methyl-D-aspartate (NMDA) receptor-rich glutamatergic neurons in the CORT-induced hippocampus. Changes in the number and composition of spines following PEA treatment could play a role in synaptic alterations in CORT-induced mice.

BDNF and its receptor TrkB have important roles in the formation of neural circuits and are widely implicated in many neuropsychiatric disorders [18]. Recently, systematic reviews and meta-analyses of clinical studies suggest that BDNF is directly involved in the pathology of depression and that the restoration of BDNF may underlie the therapeutic efficacy of antidepressant treatment [52,53]. CORT exposure reduces BDNF mRNA and protein expression in the hippocampus but not the PFC [29,43]. Moreover, CORT regulates the mRNA expression of TrkB but not NT-3 or TrkC in the hippocampus [54]. Consistently, we showed that CORT decreased the mRNA and protein levels of BDNF and TrkB in the hippocampus. However, treatment with PEA restored BDNF and TrkB expression levels in the hippocampus. BDNF/TrkB signaling increases dendritic spine density in hippocampal CA1 pyramidal neurons [55]. BDNF in the hippocampus plays a key role in chronic unpredictable mild stress (CUMS)-induced depression-like behaviors and alterations in dendritic spines in hippocampal pyramidal neurons [56]. Collectively, our results show that the inactivation of BDNF/TrkB signaling is an essential mechanism by which CORT mediates spine alterations. Furthermore, treatment with PEA restores dendritic spine architectures via the activation of BDNF/TrkB/CREB signaling in the CORT-induced hippocampus.

Chronic CORT exposure induces depression-like behavior in rodents [57]. Anhedonia, which refers to loss of interest or pleasure, is a frequent symptom of major depression in humans and is associated with dysfunction of the reward system [58]. The administration of CORT decreased the preference for the sucrose solution in mice [59]. We also found that CORT exposure decreased the preference for the sucrose solution in mice, but the administration of PEA significantly increased sucrose intake back to baseline levels. In addition, despair is another main symptom of depression, and the FST and TST are thought to analyze the despair aspect of depression-like behavior in rodents [60,61]. We found that CORT exposure significantly increased the immobility time in the FST and TST, a phenomenon that has been previously described by several papers [62,63]. Treatment with PEA significantly reduced the immobility time of mice in both tests. Likewise, treatment with PEA significantly increased climbing and swimming in the rats [64]. Depression and anxiety are different disorders, but they commonly occur together. Moreover, anxiety may occur as a symptom of major depression. CORT exposure reduces open arm exploration in the EPM, indicating increased anxiety in the rodent [65,66]. Consistently, Huanglian-Jie-Du-Tang extract ameliorates depression-like behaviors via BDNF-TrkB-CREB Pathway [67]. In addition, The VGF-derived peptide TLQP62 produces antidepressant-like effects in mice via regulation of the BDNF/TrkB/CREB signaling [68]. Similarly, we found that the administration of CORT leads to anxiety-like behavior in mice, but treatment with PEA reversed this anxiety behavior. Overall, our findings suggest that PEA is an effective treatment for the behavioral aspects of depression.

## 4. Materials and Methods

### 4.1. Reagents

The corticosterone (CAS number: 50-22-6, catalog number: 27,840, Sigma Aldrich, St. Louis, MO, USA) and the PEA (CAS number: 64-04-0, catalog number: W322008, Sigma Aldrich, St. Louis, MO, USA) were more than 99% pure and dissolved in dimethyl sulfoxide (DMSO) (CAS number: 67-68-5, catalog number: D8418, Sigma Aldrich, St. Louis, MO, USA).

### 4.2. Animals and Housing Conditions

Mice (C57BL/6N) were purchased from the Orient Bio Inc. (Seoul, Korea). The animals were housed 5 mice per cage under the condition of the temperature (23 ± 3 °C) and humidity (30–70%) with standard rodent chow and water available ad libitum and were maintained on a 12 h light/dark cycle (lights on at 8:00 a.m.). The experimental procedure was approved by the Institutional Animal Care and Use Committee at the Korea Institute of Toxicology and met National Institutes of Health guidelines for the care and use of laboratory animals (KIT-IACUC; Approval Number 1910-0333 and 1910-0332, Approval Date 1 October 2019).

### 4.3. Primary Neuronal Cultures

Primary neuronal culture was described previously [69,70]. In brief, hippocampus from E18 mice were isolated and dissociated with trituration after trypsin/EDTA treatment. Then, the cells were plated onto poly-D-lysine/laminin-coated coverslips and cultured in the medium containing neurobasal medium, 5% serum, B27 and N2 supplements. Cultured hippocampal neurons transfected with a GFP plasmid at DIV 10. After 4 days, neuronal cultures were administrated with the CORT (50 μM) for 24 h followed by treatment with PEA (100 μM) for 24 h.

### 4.4. Cell Transfection

Neuronal transfection was performed as described in previous papers [71,72]. DNA constructs were transfected into attached cells using lipofectamine (Thermo Fisher Scientific Inc., Waltham, MA, USA) according to the manufacturer’s protocol. According to the manufacturer’s instructions, per well of a 24-well culture plate, 1 μL of Lipofectamine 2000 was diluted in 50 μL of Neurobasal medium without supplements at room temperature and 5 min later was combined with 1μg of GFP-tagged plasmid (pSUPER-Venus) in 50 μL of Neurobasal medium without supplements. Incubation continued for 20 min at room temperature, and the mixture was applied to culture wells and changed in the medium containing neurobasal medium, 5% serum, B27 and N2 supplements after 6 h.

### 4.5. Reverse Transcription RCR

RNA was extracted from cultured neurons using TRIZOL reagent (Thermo Fisher Scientific Inc. Waltham, MA, USA), and cDNA was synthesized from 1 μg of total RNA using oligo-dT and random hexamers using the Verso cDNA synthesis kit (Thermo Fisher Scientific Inc. Waltham, MA, USA). PCR was performed using 1 μL of cDNA and the Master Mix (Promega Corporation, Madison, WI, USA). The sequences of the primers used were BDNF forward 5′-GCGGCAGATAAAAAGACTGC-3′ and reverse 5′-CCCGAACATACGATTGGGTA-3′, TrkB forward 5′-TGGTGCATTCCATTCACTGT-3′ and reverse 5′-CTTGGCCATCAGGGTGTAGT-3′, GAPDH forward 5′-AAGGTCATCCCAG AGCTGAA-3′ and reverse 5′-AGGAGACAACCTGGTCCTCA-3′. All primers were initially tested for their specificity by running RT-PCR samples on an agarose gel. Glyceraldehyde 3-phosphate dehydrogenase (GAPDH) was used as an internal control to normalize band intensity.

### 4.6. Immunoblotting

Western blotting was performed as described previously [73]. Tissue lysates from the hippocampal region were prepared using RIPA buffer and the sample was centrifuged at 12,000 rpm for 10 min at 4 °C. The supernatant was then collected and protein content was determined by Pierce BCA Protein Assay Kit (Thermo Fisher Scientific Inc., Waltham, MA, USA) following the manufacturer’s protocol. Proteins were separated on 8%, 10%, or 15% SDS-PAGE gradient gel and transferred onto PVDF transfer membrane (Thermo Fisher Scientific Inc., Waltham, MA, USA). Then the membrane was incubated with rabbit anti-BDNF (ab97959, Abcam, Cambridge, UK), rabbit anti-TrkB (ab23345, Abcam, Cambridge, UK), rabbit anti-CREB (#9197, Cell Signalling Technology, Danvers, MA, USA), rabbit anti-p-CREB (#9198, Cell Signalling Technology, Danvers, MA, USA), rabbit anti-Synaptophysin (SYP, ab32594, Abcam, Cambridge, UK), rabbit anti-PSD95 (ab18258, Abcam, Cambridge, UK) and mouse anti-β-actin (A5316, Thermo Fisher Scientific Inc, Waltham, MA, USA) at 4 °C overnight. Appropriate secondary antibodies conjugated to HRP were used (Thermo Fisher Scientific Inc., Waltham, MA, USA) and the ECL reagents (Thermo Fisher Scientific Inc., Waltham, MA, USA) were used for immunodetection. For quantification of band intensity, blots from *n* = 3 mice, where each three independent experiments for each molecule of interest were used. Signals were measured using ImageJ software and represented by relative intensity versus control. β-actin was used as an internal control to normalize band intensity.

### 4.7. Immunostaining

Immunostaining of dissociated neural cells was performed as described previously [74,75]. The following primary antibodies were used: Chicken anti-GFP (A10262; Thermo Fisher Scientific Inc., Waltham, MA, USA), rabbit anti-GFP (A11122; Thermo Fisher Scientific Inc., Waltham, MA, USA). Appropriate secondary antibodies conjugated with Alexa Fluor dyes (A11039, A11008; Thermo Fisher Scientific Inc., Waltham, MA, USA) were used to detect primary antibodies.

### 4.8. Morphometry

Dendritic spine density analysis was performed as previously described [40]. Briefly, primary hippocampal neuron cultures (DIV 10) were transfected with pSUPER-Venus vector for visualization. The number of dendritic spines was evaluated at DIV 16. The fluorescent images were acquired with an FV3000 confocal microscope (Olympus life science, Tokyo, Japan), and the settings were kept consistent for all samples. The dendritic spines were counted on segments of secondary dendrites which are 50–100 μm apart from the center of the cell soma by an investigator blinded to the groups. Dendritic spines can be classified into categories such as filopodia, thin, mushroom or stubby spines. Filopodia are typically longer (>2 μm) and normally have no clear head; thin spines have a thin, long neck (>1 μm) and small heads; mushroom spines have a short and narrow neck (<1 μm) and a large head (>0.6 μm), whereas the stubby spines have a head but no neck.

### 4.9. Sucrose Preference Test (SPT)

The sucrose preference test was performed as described previously [47] to assess anhedonia in mice. This test was carried out prior to the start of CUMS and at the end of CUMS. Mice were kept individually in separate cages and were allowed to adapt to two bottles of solution (filled with 1.0% sucrose solution) for 24 h. For the next 24 h, one bottle of sucrose solution was replaced with water. Then, the mice were subjected to 24 h of food and water deprivation, followed by exposure to two pre-weighed bottles of solution (1.0% sucrose solution and plain water, respectively) for 1 h. The position of the bottles was switched for each trial. After the test, the weight of sucrose solution and water consumed was recorded. Sucrose preference was calculated as a ratio of the weight of sucrose solution consumption to the weight of total fluid intake, SP = [sucrose preference/(sucrose intake + water intake)] × 100%.

### 4.10. Tail Suspension Test (TST)

The total duration of immobility induced by tail suspension test was measured according to the method of [76]. Mice both acoustically and visually isolated were suspended 50 cm above the floor by adhesive tape placed approximately 1 cm from the tip of the tail. Immobility time was recorded during a 5 min test.

### 4.11. Forced Swimming Test (FST)

Forced swimming test was performed as described previously [77] to assess the anxiety-related behavior in mice. A vertical glass cylinder (20 cm height, 20 cm in diameter) was filled with 25 ± 1 °C water to a depth of 30 cm. For testing, each mouse was placed in the cylinder for 5 min, and duration of immobility, swimming, and climbing were scored. Water in the cylinder was changed for each mouse. Immobility was recorded whenever animals stopped swimming and remained floating in the water, with their heads above the surface.

### 4.12. Elevated Plus Maze Test

The elevated plus maze test was performed as described previously [78] to assess the anxiety-related behavior in mice. In the EPM test for mice, two opposite open arms (25 cm × 5 cm) and two opposite closed arms (25 cm × 5 cm × 16 cm) connected by a central square (5 × 5 cm) make up the apparatus, which is located 50 cm above the floor. The mice were individually placed in the central zone facing one of the open arms and a video camera mounted above the maze connected to a computer was used to monitor and score behavior during a 5-min experimental period. Testing sessions were filmed using a digital camera (SLA-3580DN, Samsung Techwin Co., Ltd., Seoul, Korea) and later analyzed with EthoVision XT software (Version 14.0, Noldus Information Technology Inc., Leesburg, VA, USA).

### 4.13. Statistical Analysis

Normal distribution was tested using the Kolmogorov–Smirnov test, and variance was compared. Unless otherwise stated, statistical significance was determined by one-way or two-way analysis of variance (ANOVA) followed by the Bonferroni post hoc test for multiple comparisons. Data were analyzed using GraphPad Prism (Version 8.0.x, GraphPad Software, Inc., La Jolla, CA, USA) and presented as mean (±) SEM. P values were indicated in figure legends.

## 5. Conclusions

We conclude from the present study that PEA ameliorates depression-like symptoms in a CORT-induced model of depression. PEA ameliorates depression-like behaviors by regulating dendritic spine architecture and synaptic function in the hippocampus. The rescue of these depression-like behaviors and abnormal synaptic function may be due to changes in BDNF/TrkB signaling, which are downregulated in response to CORT exposure. However, treatment with PEA or a traditional antidepressant restores BDNF/TrkB/CREB signaling. This study provides insight into the pathophysiology of depression and suggests that PEA could be useful in treating depression disorders.

## Supplementary Materials

Supplementary materials can be found at https://www.mdpi.com/1422-0067/21/23/9103/s1.

## Abbreviations

PEA2	Phenylethylamine
CORT	Corticosterone
MDD	Major depressive disorder
HPA	Hypothalamic–pituitary–adrenal
BDNF	Brain-derived neurotrophic factor
TrkB	Tropomyosin receptor kinase B
Ras	Small GTP-binding protein
ERK	Extracellular-signal-regulated kinase
PLC-γ	Phosphoinositide phospholipase C
PI3K	Phosphatidylinositol 3-kinases
AKT	Serine/threonine kinase
TAAR	Trace amine-associated receptor
SYP	Synaptophysin
PSD95	Postsynaptic density protein 95
E/I	Excitatory/inhibitory
SPT	Sucrose preference test
TST	Tail suspension test
EPM	Elevated plus-maze
FST	Forced swimming test
NMDA	N-methyl-D-aspartate receptor
NT3	Neurotrophin-3
TrkC	Tropomyosin receptor kinase C
