# Supplementary material for: 2-Phenylethylamine (PEA) Ameliorates Corticosterone-Induced Depression-Like Phenotype via the BDNF/TrkB/CREB Signaling Pathway
Source: Int J Mol Sci. 2020 Nov 30;21(23):9103. doi: 10.3390/ijms21239103 (PMC7729630; doi:10.3390/ijms21239103)
Supplement: Supplementary file 1 [file ijms-21-09103-s001.pdf]

Supplementary Information

Title: 2-Phenylethylamine (PEA) ameliorates corticosterone-induced depression-like phenotype via the BDNF/TrkB/CREB signaling pathway

Young-Ju Lee [1,3,#], Hye Ryeong Kim[1,4,#], Chang Youn Lee[1,#], Sung-Ae Hyun[1], Moon Yi Ko[1], Byoung-Seok Lee[2], Dae Youn Hwang[3] and Minhan Ka[1,*]

Supplementary figure legends

Supplementary Figure 1. Effect of CORT and PEA on cultured hippocampal neuron viability (A, B) The survival ratios of cultured hippocampal neurons after CORT **(A)** and PEA **(B)** treatment for 24 h. Statistical significance was determined by two-way ANOVA with Bonferroni correction test. Data are shown as relative changes versus controls. $^{*}p < 0.05$, $^{**}p < 0.01$, $^{***}p < 0.001$.

Supplementary Figure 1.

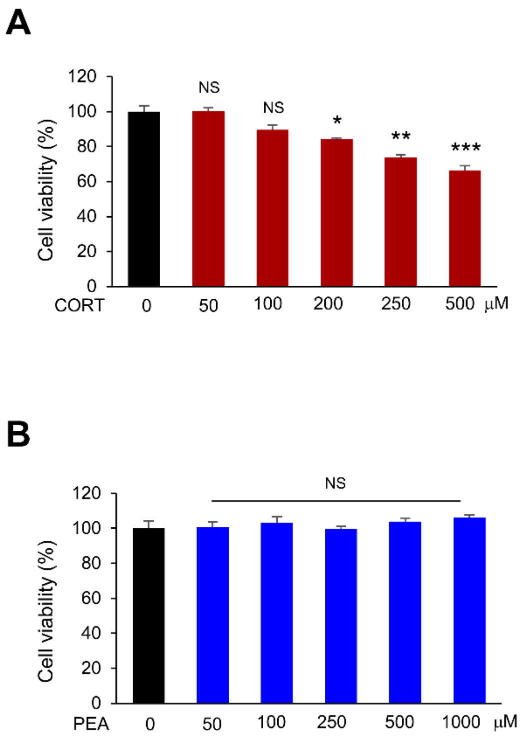
A
Cell viability (%)
120
100
80
60
40
20
0
NS
NS
*
**
***
CORT
0
50
100
200
250
500
μM
B
Cell viability (%)
120
100
80
60
40
20
0
NS
PEA
0
50
100
250
500
1000
μM
